# Integrating DFT Computations and QSAR Modeling to Predict Adsorption of Organic Pollutants onto Microplastics in Aqueous Environments

**DOI:** 10.3390/ma19071403

**Published:** 2026-04-01

**Authors:** Ya Wang, Chao Li, Honghong Yi, Xiaolong Tang, Peng Zhao

**Affiliations:** 1 School of Energy and Environmental Engineering, University of Science and Technology Beijing, Beijing 100083, China; 2 Engineering Laboratory for Water Pollution Control and Resources Recovery, State Environmental Protection Key Laboratory of Wetland Ecology and Vegetation Restoration, School of Environment, Northeast Normal University, Changchun 130117, China

**Keywords:** microplastics, adsorption energy, aqueous environment, density functional theory computation, quantitative structure activity relationship

## Abstract

Understanding the adsorption of organic pollutants onto microplastics in aqueous environments is crucial for assessing their environmental behavior and ecological risks. Herein, we used density functional theory (DFT) computations to simulate the aqueous adsorption of 54 organic compounds onto three representative microplastics, namely polyethylene (PE), polyoxymethylene (POM), and polyvinyl alcohol (PVA). Afterwards, based on theoretical molecular structural descriptors, we developed six quantitative structure activity relationship (QSAR) models based on datasets of 43 and 54 organic compounds, respectively. The results demonstrated that the oxygen-containing POM and PVA microplastics exhibited weaker adsorption in the aqueous phase compared to that in the gas phase. Furthermore, it revealed that the electron-rich atoms, van der Waals volumes and molecular polarizability exert substantial effects on the adsorption process on microplastics in water. These robust QSAR models can enable the prediction of adsorption energies for various organic pollutants on microplastics, which can offer a rapid approach for generating adsorption data. Moreover, the insights into adsorption mechanisms can provide a theoretical basis for designing modified or alternative plastics with lower environmental risks.

## 1. Introduction

Plastics have been widely used in various fields, including packaging [1], agriculture [2], construction and automotive manufacturing [3], which leads to a continuous increase in their production and consumption. The global plastic market has reached a valuation of $645.55 billion in 2025, and will further expand to $799.24 billion by 2030 [4]. This increase in the global plastic production and consumption has inevitably resulted in a dramatic accumulation of plastic waste. It is estimated that between 19 and 23 million tonnes of plastic waste are released into aquatic environments annually [5]. The plastic waste entering the environment is gradually fragmented and aged into microplastics (MPs) (with sizes ranging from 1 μm to 5 mm) [6] through ultraviolet radiation, mechanical abrasion, or biological action. Microplastics have been frequently detected in diverse aquatic environments globally [7]. Owing to their strong hydrophobicity, microplastics in aquatic environments tend to adsorb organic pollutants, leading to combined pollution. Moreover, microplastics can act as vectors [8], influencing the transport, transformation, and bioavailability of organic pollutants, thereby altering their ecological risks. Therefore, investigating the adsorption of organic pollutants onto microplastics in aqueous environments is of great significance for assessing their ecological risks. Furthermore, elucidating their adsorption behavior can also provide a scientific basis for designing green alternatives to plastic products.

The adsorption of some different organic pollutants, e.g., polycyclic aromatic hydrocarbons [9], phenols [10], and biphenyl [11], on various microplastics have been explored through adsorption isotherms and adsorption kinetics experiments in previous studies. The results implied that different interactions including hydrophobic interactions [12], hydrogen bonding [13,14], π–π interactions [15,16] and electrostatic interactions [17] may simultaneously contribute to the adsorption of organic pollutants onto MPs. As for the same neutral organic compounds, polar MPs (polybutylene succinate and polycaprolactone) exhibited stronger adsorption capabilities than nonpolar MPs (low-density polyethylene and polystyrene) [18]. This may be attributed to the presence of polar interactions in addition to hydrophobic interactions, which facilitates the adsorption of organic pollutants onto polar microplastics. It should be noted that the adsorption properties of microplastics may also undergo changes after aging. For example, aged microplastics exhibit enhanced adsorption capabilities for hydrophilic pharmaceuticals and antibiotics, which may be attributed to factors such as surface oxidation [19,20]. However, for 2,2′,4,4′-tetrabromodiphenyl ether, the aged polystyrene (PS) microplastics showed a lower adsorption capacity compared to the pristine PS MPs [21]. Given the wide variety of MPs and the changes in their surface morphology and chemical properties including functional groups and specific surface area after aging, it remains a significant technical challenge to ensure complete uniformity in the types and quantities of functional groups of MPs in experiments.

Unlike experimental methods, theoretical computation including density functional theory (DFT) method [22,23] allows for constructing MPs models with well-defined structures and functional groups under ideal conditions, thereby ensuring the uniformity of MPs. Furthermore, DFT computations can provide atomic-level insights, including adsorption geometries, charge distribution, and electrostatic potential, which are crucial for a deeper understanding of the adsorption mechanisms onto MPs. For example, Wang et al. [24] examined the adsorption of tetracycline onto MPs with the DFT method. The calculated electrostatic potential results showed that the phenolic hydroxyl hydrogen of tetracycline delivered the positive potential which may have allowed it to interact with MPs through hydrogen bonds. Tang et al. [25] investigated the adsorption of seven different organic pollutants on polystyrene nanoplastics through DFT simulations, and found that phenanthrene was prone to be adsorbed on polystyrene nanoplastics in a parallel orientation. However, considering the large amount of organic pollutants in the environment, it is impractical to conduct DFT computations for each pollutant–microplastic pair due to the excessive computational cost and time. To address this, there is an urgent need to develop predictive models so as to achieve the high-throughput estimation of adsorption onto MPs.

Quantitative structure activity relationship (QSAR) technology [26] has been used for developing predictive models on the basis of the relationships between molecular structural descriptors and adsorption data, enabling the high-throughput prediction of adsorption by MPs. Several predictive models have been established for estimating the adsorption of organic pollutants on distinct MPs [15,18,27,28,29,30,31,32,33]. Current QSAR models are primarily focused on predicting adsorption equilibrium constants. In addition, most molecular structural descriptors in these predictive models are derived from experimental determination. Consequently, the application of these predictive models is limited by the availability of experimental data. As for the adsorption energy, a parameter that intuitively describes the interactions between organic pollutants and microplastics at the molecular level and new predictive models need to be established. Therefore, it is necessary to develop QSAR models based on theoretically derived molecular structural descriptors for predicting the adsorption energies between organic pollutants and MPs.

In this study, density functional theory (DFT) computations were performed to explore the adsorption of 54 organic pollutants with different functional groups onto three types of MPs, i.e., polyethylene (PE), polyoxymethylene (POM), and polyvinyl alcohol (PVA), in aqueous environments. Based on the adsorption energies derived from DFT calculations along with the theoretical molecular structural descriptors, three QSAR prediction models were developed with the split training set consisting of 43 organic compounds. These three QSAR models were evaluated in terms of their goodness of fit, robustness, and predictive capability. Furthermore, another three QSAR models were developed on the basis of the total 54 organic compounds. The applicability domains of these six QSAR models were characterized with the Williams plots. These models developed herein can facilitate the high-throughput estimation of the aqueous adsorption energies for organic pollutants on PE, POM and PVA MPs.

## 2. Computational Methods

### 2.1. Microplastics and Organic Compounds

**Figure 1:**
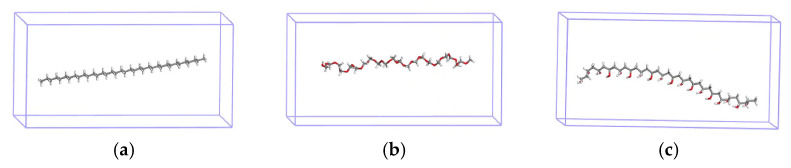
(**a**) PE, (**b**) POM and (**c**) PVA MPs.

Polyethylene (PE), polyoxymethylene (POM), and polyvinyl alcohol (PVA) MPs were used as MP models (Figure 1); these models showed great adsorption capabilities in our previous study [34]. Considering the trade-off between computational cost and model representativeness, we selected short-chain polymers as models for microplastics. To ensure that these three MPs models have comparable lengths and are substantially greater than the molecular structures of organic compounds, the PE and PVA models each consist of 18 repeating units, whereas the POM model contains 22 repeating units. Furthermore, the ends of the short-chain polymer models were saturated with hydrogen atoms to avoid dangling bonds. A vacuum region larger than 15 Å was introduced along both the *x* and *y* directions. The dimensions of the periodic box (25 Å × 25 Å × 50 Å) are sufficiently large to prevent interactions between neighboring mirror images. A total of 54 organic compounds with distinct functional groups were also constructed. They are composed of 40 representative aliphatic and aromatic compounds, six brominated flame retardants, and eight phosphorus-based flame retardants (Table 1). All the MPs (PE, POM and PVA) models and 54 organic compounds were optimized via DFT.

### 2.2. Density Functional Theory Simulations

The DMol^3^ program [35,36,37] was employed for all DFT computations. The generalized gradient approximation (GGA) with the Perdew–Burke–Ernzerhof (PBE) functional was adopted [38,39], together with a double-numerical basis set augmented with polarization functions (DNPs) [40,41]. Moreover, to better describe long-range interactions, the Grimme van der Waals correction (PBE+D2) [42,43] was also utilized. The PBE+D2 has also been validated for investigating the adsorption onto atmospheric nanoplastics [34]. The aqueous environment was implicitly simulated using the conductor-like screening model (COSMO) [44], where the dielectric constant of water was set to 78.54. Within the COSMO formalism, the solvent water is modeled as a dielectric continuum surrounding a cavity with the shape of the water molecule. The electrostatic interactions between the solute and water are quantified through cavity surface charges, which can be calculated from the electrostatic potentials. It offers a more refined technique compared to alternative solvent reaction field approaches [45]. However, it also has limitations, such as the inability to observe hydrogen bonding as explicitly as with water molecules. The favorable adsorption configurations were identified through the sorption module of Materials Studio 8.0, as described in our previous research [46]. These favorable adsorption configurations were further optimized via DFT. The convergence threshold for electronic energy was set to 2.0 × 10^−5^ Ha, and the force convergence criterion was set to 0.004 Ha/Å. Hirshfeld charge analysis was also performed for obtaining the charge distribution.

### 2.3. Adsorption Energies (E_ad_) and Theoretical Molecular Structural Descriptors

Based on the total energies obtained from DFT computations, the adsorption energy can be calculated using the following equation:

*E*_ad_ = *E*_MPs+com_
*− E*_MPs_ − *E*_com_ (1)

where *E*_MPs+com_ is the total energy of the complex consisting of MPs and organic compounds; *E*_MPs_ represents the total energy of MPs and *E*_com_ denotes the total energy of the organic compound.

The theoretical molecular structural descriptors of 54 organic compounds were calculated with PaDEL software (version 2.21) [47]. Before calculating the theoretical molecular descriptors, we first optimized the geometries of these compounds using the DFT method. Subsequently, 2325 theoretical molecular descriptors and fingerprints were calculated with these optimized structures. As in our previous study [34], after eliminating descriptors with zero values, 643 descriptors were retained for QSAR model development. Subsequently, stepwise multiple linear regression (MLR) analysis was conducted to further screen the descriptors and build predictive models. All the retained descriptors exhibited variance inflation factor (*VIF*) values below 10 [48].

### 2.4. Model Development and Characterization

A total of 54 organic compounds were initially randomly split into a training set and a validation set at a ratio of 4:1. The training set comprised 43 compounds for model development, while the validation set consisted of 11 compounds for model validation. MLR analysis was employed to develop the QSAR models. The goodness of fit, robustness, and predictive performance of the models were evaluated using the determination coefficient (*R*^2^), root mean square error (*RMSE*), leave-one-out cross-validated *Q*^2^ (*Q*^2^_LOO_) and the external explained variance *Q*^2^ (*Q*^2^_ext_). In addition, y-randomination was also used for evaluating the robustness of the developed predictive models. Considering that the applicability domain (AD) is inherently dependent on the training set, three additional models were subsequently developed using the 54 organic compounds. The ADs of these six QSAR models were characterized by Williams plots, generated by plotting the standardized residuals (δ*) against leverage values (*h*) [49]. The warning leverage (h*) was calculated as 3(*k* + 1)/*n*, where *k* is the number of descriptors and *n* is the number of organic compounds in the training set. A compound with an absolute value of δ* greater than 3 will be considered a potential outlier. If the *h* value of a compound in the training set exceeds the h* value, it indicates that this compound has significant influences on the predictive model. On the other hand, if the *h* value of a compound in the validation set exceeds the h* value, it suggests that the predicted value for this compound is an extrapolation of this model.

## 3. Results and Discussion

### 3.1. Adsorption Energy (E_ad_) Values for Organic Compounds on MPs

**Figure 2:**
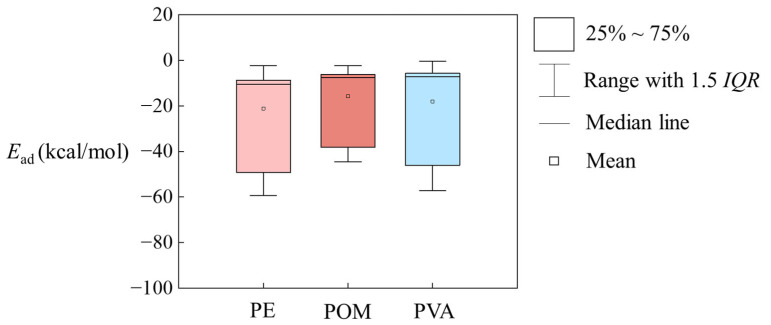
Box–whisker plot for *E*_ad_ values on PE, POM and PVA MPs in the aqueous environment (*IQR* = the third quartile − the first quartile).

The adsorption energies (*E*_ad_) for 54 organic compounds on PE, POM and PVA MPs in aqueous environment derived from DFT calculations are listed in Table 1, which shows that these *E*_ad_ values range from −0.36 to −59.28 kcal/mol. Figure 2 illustrates that the average *E*_ad_ values of organic compounds on PE, POM and PVA MPs are comparable. Specifically, the *E*_ad_ ranges are −2.33~−59.28 kcal/mol for PE MPs, −2.31~−44.49 kcal/mol for POM MPs, and −0.36~−57.25 kcal/mol for PVA MPs. Notably, the *E*_ad_ values for the 14 emerging pollutants including six brominated flame retardants, and eight phosphorus-based flame retardants on PE, POM and PVA MPs are more negative than −40 kcal/mol. It implies that the introduction of Br atoms and -PO_4_ groups significantly enhances the adsorption onto MPs. In addition, the adsorption equilibrium configurations and the distances between the center of the molecule and the nearest carbon atom in PE, POM or PVA are shown in Table S1. The distance of organic compounds on PE MPs is in the range of 3.284~4.482 Å; on POM MPs, it is 3.193~5.424 Å; and on PVA MPs, it is 3.407~5.499 Å.

Note that we have previously conducted DFT computations to investigate the adsorption of some representative organic compounds on PE, POM and PVA MPs in the gaseous phase, and found that the functional groups had effects on the adsorption energies [34]. Herein, we compared the adsorption energies for 15 organic compounds on PE, POM and PVA MPs under aqueous and gaseous environments in Table S2. The results indicated that the adsorption trends of different organic compounds on the PE MPs vary between the gaseous and aqueous phases. For adsorption on PE MPs, formic acid, malonic acid, aniline, phenol, and benzyl alcohol demonstrated lower adsorption energies in the aqueous than in the gaseous phase. This may be because the five compounds all contain strongly polar O-H or N-H bonds, which readily form electrostatic interactions with polar water molecules, resulting in steric hindrance that impedes their access to the microplastics. Conversely, the remaining ten organic compounds displayed higher adsorption energies in the aqueous environment compared to the gaseous phase. However, the adsorption energies of these 15 organic compounds onto POM and PVA MPs are lower in aqueous environments compared to in gaseous environments. This phenomenon can be attributed to the presence of oxygen atoms in both POM and PVA MPs, which readily generate electrostatic interactions with water molecules. Consequently, the available adsorption sites on these microplastics are reduced, hindering the adsorption of organic pollutants and thus resulting in a weaker apparent adsorption energy. These results imply that incorporating oxygen atoms into plastic may suppress their affinity for organic pollutants, consequently reducing the potential for combined pollution.

### 3.2. QSAR Models Based on 43 Organic Compounds for Predicting E_ad_ Values on PE, POM, and PVA MPs in Aqueous Environments

We established three QSAR models based on the training set to predict the *E*_ad_ values on PE, POM, and PVA MPs under aqueous environments. These models are described below.

In terms of PE MPs, the model is as follows:

*E*_ad_ = 9.879 − 0.039 × *ATSC1m* − 0.212 × *AATSC0v* − 21.91× *MATS1m* − 0.638 × *BCUTw-1h* (2)

*n_t_* = 43, *R*^2^ = 0.98, *RMSE*_t_ = 2.44, *Q*^2^_LOO_ = 0.98, *F* = 569.27, *p* < 0.001, *n*_v_ = 11, *R*^2^_ext_ = 0.92, *Q*^2^_ext_ = 0.85, *RMSE*_V_ = 7.82.

In terms of POM MPs, the model is as follows:

*E*_ad_ = 10.889 − 0.04 × *ATSC1m* − 0.217 × *AATSC0v* − 3.728 × *MATS1m* − 0.482 × *BCUTw-1h* (3)

*n_t_* = 43, *R*^2^ = 0.96, *RMSE*_t_ = 3.11, *Q*^2^_LOO_ = 0.94, *F* = 212.516, *p* < 0.001, *n*_v_ = 11, *R*^2^_ext_ = 0.88, *Q*^2^_ext_ = 0.81, *RMSE*_V_ = 6.59.

In terms of PVA MPs, the model is as follows:

*E*_ad_ = 10.709 − 0.047 × *ATSC1m* − 0.157 × *AATSC0v* − 6.006 × *MATS1m* − 0.661 × *BCUTw-1h* (4)

*n_t_* = 43, *R*^2^ = 0.96, *RMSE*_t_ = 3.96, *Q*^2^_LOO_ = 0.94, *F* = 217.305, *p* < 0.001, *n*_v_ = 11, *R*^2^_ext_ = 0.90, *Q*^2^_ext_ = 0.85, *RMSE*_V_ = 7.88.

**Figure 3:**
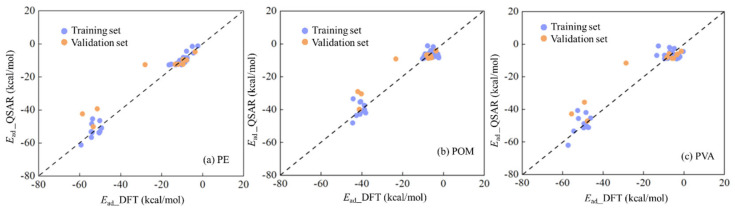
Predicted aqueous *E*_ad_ values from QSAR models established with 43 organic compounds (*E*_ad*_*_QSAR) versus those from DFT computations (*E*_ad*_*_DFT) on (**a**) PE, (**b**) POM and (**c**) PVA MPs.

Among the parameters listed above, *n_t_* refers to the number of organic compounds in the training set, while *n*_v_ denotes that in the validation set. These statistical parameters, namely *R*^2^, *R*^2^_ext_, *Q*^2^_LOO_, *Q*^2^_ext_, *RMSE*_t_, and *RMSE*_V_, were calculated for assessing the goodness-of-fit, robustness and predictive capability of these established QSAR models. All *R*^2^ and *Q*^2^ values exceed the thresholds of 0.60 and 0.50 [50], respectively, meeting the required standards. Moreover, the mean *R*^2^ values obtained from y-randomization are 0.12 (for PE MPs), 0.10 (for POM MPs), and 0.07 (for PVA MPs) (Table S3), respectively, all of which are significantly lower than those for the aforementioned three models. It further confirms that these three models are reliable and free from overfitting or chance correlation. In addition, the variance inflation factor (*VIF*) values (Table S4) for these four descriptors are all below 10 [48], implying no severe multicollinearity among these four descriptors. The values for these statistical parameters demonstrated that these three QSAR models perform satisfactorily. Moreover, we compared the predicted *E*_ad_ values derived from QSAR models and those from DFT computations in Figure 3, which exhibited that those predicted *E*_ad_ values showed strong consistency with those from DFT calculations.

**Figure 4:**
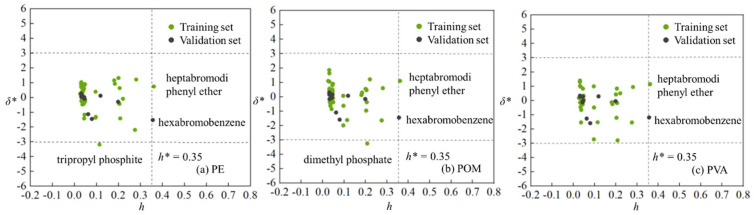
Applicability domains characterized with the Williams plot for QSAR models developed with 43 organic compounds of (**a**) PE, (**b**) POM and (**c**) PVA MPs.

To characterize the applicability domains (ADs), we further calculated the standardized residuals (δ*) and leverage values (*h*) for the Williams plot. As shown in Figure 4, for the QSAR predictive model of adsorption on PE MPs, the standardized residual of tripropyl phosphite was slightly below −3, identifying it as an outlier. In the QSAR model for adsorption on POM, the standardized residual of dimethyl phosphate also fell outside the range of −3 to 3, marking it as an outlier. It should be noted that the *h* value of one compound in training set, i.e., heptabromodiphenyl ether, marginally exceeded the warning value (0.35). However, the *E*_ad_ values for heptabromodiphenyl ether on PE, POM and PVA MPs predicted by the three QSAR models were in good agreement with those deverived from DFT computations. This suggests that heptabromodiphenyl ether may have significant influences on the QSAR models. In addition, the *h* value of one compound from the validation set, namely hexabromobenzene, was also slightly above the warning value (0.35), yet the *E*_ad_ values predicted by the three QSAR models corresponded well with those calculated ones from DFT. This indicates that the established QSAR models possess good extrapolation capability. Consequently, these three QSAR models presented herein can be reliably applied to estimate the adsorption of diverse organic pollutants onto PE, POM, and PVA MPs in aquatic environments.

### 3.3. QSAR Models Based on 54 Organic Compounds for Predicting E_ad_ Values on PE, POM, and PVA MPs in Aqueous Environments

Given that the applicability domain of a predictive model is inherently linked to the organic compounds employed in its development, we further constructed three additional QSAR models using the entire dataset, which are presented as follows.

Regarding PE MPs, the model is as follows:

*E*_ad_ = 11.520 − 0.090 × *AATS1m* + 6.934 × *AATS7p* − 4.129 × *ATSC0p* + 28.365 × *AATSC1p* (5)

*n* = 54, *R*^2^ = 0.96, *Q*^2^_LOO_ = 0.95, *RMSE* = 3.91, *F* = 285.277, *p* < 0.001.

Regarding POM MPs, the model is as follows:

*E*_ad_ = 9.285 − 0.068 × *AATS1m* + 6.050 × *AATS7p* − 3.229 × *ATSC0p* + 31.582 × *AATSC1p* (6)

*n* = 54, *R*^2^ = 0.95, *Q*^2^_LOO_ = 0.93, *RMSE* = 3.47, *F* = 220.548, *p* < 0.001.

Regarding PVA MPs, the model is as follows:

*E*_ad_ = 14.772 − 0.097 × *AATS1m* + 6.944 × *AATS7p* − 3.987 × *ATSC0p* + 36.499 × *AATSC1p* (7)

*n* = 54, *R*^2^ = 0.94, *Q*^2^_LOO_ = 0.92, *RMSE* = 4.81, *F* = 190.469, *p* < 0.001.

**Figure 5:**
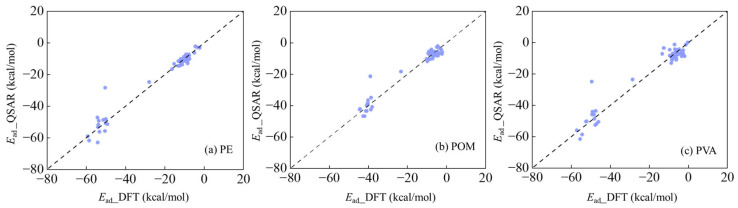
Predicted aqueous *E*_ad_ values from QSAR models established with 54 organic compounds (*E*_ad*_*_QSAR) versus those from DFT computations (*E*_ad*_*_DFT) on (**a**) PE, (**b**) POM and (**c**) PVA MPs.

Here, *n* represents the number of organic compounds used for establishing the QSAR predictive models. Analogously to the three preceding models, statistical parameters including *R*^2^, *Q*^2^_LOO_ and *RMSE* were also calculated to evaluate the goodness-of-fit and robustness of the model. The calculated *R*^2^ and *Q*^2^_LOO_ values all exceed the thresholds of 0.60 and 0.50, respectively [50], satisfying the required benchmarks. Furthermore, a y-randomization test was also performed for excluding the possibility of chance correlation. The obtained mean *R*^2^ values for the permuted datasets are 0.09 (for PE MPs), 0.09 (for POM MPs), and 0.05 (for PVA MPs) (Table S3), which are substantially lower than those of these three original models. This finding provides strong evidence that these models are robust, and not based on spurious correlations. Moreover, the *VIF* values also fall below 5 (Table S4) [48], suggesting that multicollinearity is not a significant concern in these QSAR models. These statistical measures collectively demonstrate the satisfactory performance of the three QSAR models. Additionally, as shown in Figure 5, the *E*_ad_ values predicted by these three QSAR models align closely with those derived from DFT computations. Thus, these QSAR models can be used for the high-throughput prediction of the *E*_ad_ values for different organic pollutants on PE, POM and PVA MPs in the aqueous environment.

**Figure 6:**
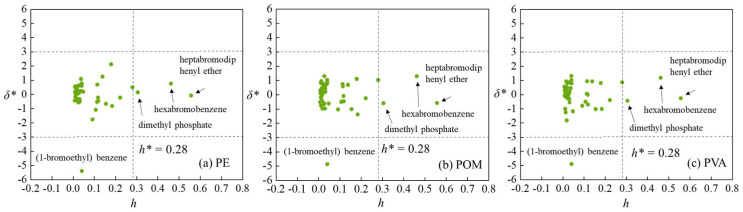
Applicability domains characterized with the Williams plot for QSAR models developed with 54 organic compounds of (**a**) PE, (**b**) POM and (**c**) PVA MPs.

Similarly, δ* and *h* values were also calculated so as to generate Williams plots defining the applicability domains of these three QSAR models. It can be observed from Figure 6 that, for these three QSAR models predicting adsorption on PE, POM, and PVA MPs, although the *h* value of (1-bromoethyl) benzene was below the warning value of 0.28, its δ* values were all less than −3. This indicates that (1-bromoethyl) benzene is an outlier. Note that it was not identified as an outlier in the three QSAR models developed with the split training set. This suggests that the different descriptors employed in our new QSAR models with the entire dataset cannot adequately characterize the structural features of (1-bromoethyl) benzene to some extent. In addition, three organic compounds, namely heptabromodiphenyl ether, hexabromobenzene and dimethyl phosphate, consistently showed *h* values higher than the warning value (0.28) across these three QSAR models, while their δ^*^ values remained within the range of −3 to 3. It implies that their structural descriptors used in the current three QSAR models are substantially distinct from those of the remaining compounds in the dataset. The *E*_ad_ values for these three organic compounds can exert strong leverage on the fitted regression line, and effectively expand the boundary of applicability domains for these three QSAR models. The absolute values of δ* for these three organic compounds are less than 3, demonstrating that these three QSAR models possess good adaptability. The current developed models cover various organic compounds with the functional groups including -Cl, -Br, -COOH, -CHO, >C=C<, -NH_2_, -OH, -NO_2_, -CN, -COO-, -C-O-C-, and P(=O)(O-)_3_.

### 3.4. Comparison Between Different Models

We have listed some predictive models for the adsorption on PE MPs in seawater, freshwater and pure water in Table 2. All these previous models can be used for predicting the logarithmic value of the adsorption equilibrium constant of organic compounds onto PE MPs, while our models are focused on predicting the adsorption energies on PE, POM and PVA MPs. Compared with previous models, our models expand the ADs to emerging contaminants such as brominated flame retardants and phosphorus-based flame retardants. However, these established models in this study only can be used for the adsorption in pure water. Therefore, future research could consider simulating adsorption in seawater and freshwater environments.

### 3.5. Interpretation of the Adsorption Mechanisms

**Figure 7:**
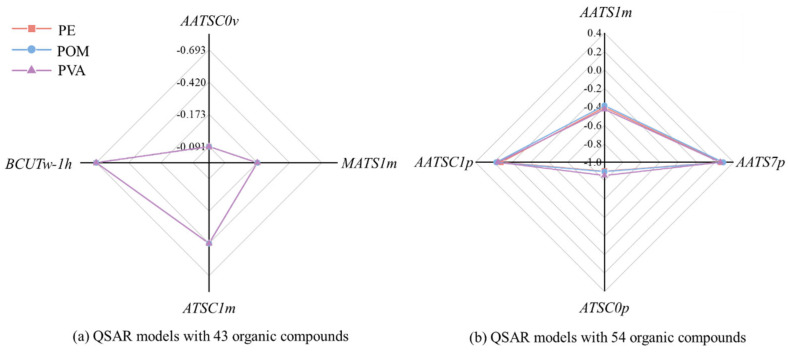
Visualization of standardized coefficients in QSAR models via radar plots.

As shown in Equations (2)–(7), the four descriptors, i.e., *ATSC1m*, *AATSC0v*, *MATS1m* and *BCUTw-1h*, used in the QSAR models based on the 43 organic compounds are different from those 4 ones (namely, *AATS1m*, *AATS7p*, *ATSC0p* and *AATSC1p*) used in the QSAR models on the basis of 54 organic compounds. This suggests that these theoretical molecular structural descriptors have distinct contributions to the prediction of *E*_ad_ values on the PE, POM and PVA MPs in aqueous environments. The radar plots (Figure 7) were employed to illustrate the differences among the standardized coefficients of these descriptors. The values for standardized coefficients, and the *VIF*, *p* and *t* values are listed in Table S4.

All the standardized coefficients for *ATSC1m*, *AATSC0v*, *MATS1m* and *BCUTw-1h* in these three QSAR models based on 43 organic compounds are negative (Figure 7a), implying a negative relationship between these four theoretical molecular structural descriptors and the *E*_ad_ values on PE, POM and PVA MPs. Compounds possessing high values for these four parameters will have more negative *E*_ad_ values, indicating that they are more prone to being adsorbed by microplastics. Among these four descriptors, *BCUTw-1h* has the most significant impact on predicting the *E*_ad_ values, shown in Figure 7. *BCUTw-1h* [51], a BCUT (Burden, CAS, and University of Texas) descriptor, represents n low highest-atom-weighted BCUTS. This reflects the connectivity and the atomic properties with atomic weight [52]. For example, the *BCUTw-1h* values for benzene, aniline and nitrobenzene are in the following order: 12.15 < 14.00 < 16.00. The more atoms rich in electrons (e.g., N and O atoms) a compound contains, the higher the *BCUTw-1h* value tends to be. A compound possessing a higher *BCUTw-1h* value tends to interact with MPs more strongly. The N and O atoms rich in electrons are prone to interacting with MPs through electrostatic interactions, which may enhance the interactions between the organic compounds and MPs. As exhibited in Figure S1, the O atom in nitrobenzene possesses −0.228 e, while the N atom in aniline possesses −0.185 e, both of which are more negative than −0.048 e on the carbon atom from benzene. Note that another two descriptors, namely *ATSC1m* and *MATS1m*, are both weighted by mass. *ATSC1m* [53] denotes Centered Broto–Moreau autocorrelation-lag1/weighted by mass, illustrating the distribution of a property in relation to the topological structures. *MATS1m* [54] is Moran autocorrelation-lag1/weighted by mass, which represents the total sum of the products of terminal atom weights, accumulated across all paths of a specific length (referred to as the lag) in the molecular graph. This is also related to the mass similarity between neighboring atoms [55]. In addition, *AATSC0v* [56] is also an autocorrelation descriptor, which denotes average centered Broto–Moreau autocorrelation-lag0/weighted by van der Waals volumes. One compound with more substituents has a smaller *AATSC0v* value. For example, 1,2-dinitrobenzene, 1,3-dinitrobenzene and 1,4-dinitrobenzene each have an *AATSC0v* value of 33.88, which is lower than the value of 44.16 for nitrobenzene. The decrease in the *AATSC0v* value will result in more negative adsorption energies, indicating stronger interactions between the organic compound and microplastics.

It should be noted that there are another four molecular structural descriptors, i.e., *AATS1m*, *AATS7p*, *ATSC0p*, and *AATSC1p*, used in the QSAR models based on 54 organic compounds. Figure 7b shows that the standardized coefficients for *ATSC0p* and *AATS1m* are negative, while those for *AATS7p* and *AATSC1p* are positive. These three descriptors, namely *ATSC0p*, *AATS7p* and *AATSC1p*, are related with molecular polarizabilities. *ATSC0p* [57] is centered Broto–Moreau autocorrelation-lag 0/weighted by polarizabilities; *AATS7p* [58] represents average Broto-Moreau autocorrelation-lag7/weighted by polarizabilities; *AATSC1p* [59] denotes average centered Broto–Moreau autocorrelation-lag1/weighted by polarizabilities. This suggests that the molecular polarizability has a significant effect on the adsorption of organic compounds by microplastics in aqueous environments. This might result from polarizability-induced changes in the interactions between organic compounds and microplastics, ultimately affecting the adsorption. In addition, *AATS1m* [60] is also a descriptor weighted by mass, denoting the average Broto–Moreau autocorrelation-lag1/weighted by mass.

Overall, the adsorption process towards microplastics in aqueous environments is significantly influenced by electron-rich atoms, van der Waals volumes and molecular polarizability. In view of this, regulating the structure of plastics can mitigate their polarizability and van der Waals volumes with organic pollutants, thereby reducing their adsorption capacity and subsequent combined pollution in the environment.

## 4. Conclusions

Herein, the adsorption energies of 54 organic pollutants onto three types of microplastic, i.e., PE, POM and PVA, in aqueous environments were estimated using DFT computations. By incorporating theoretical molecular structural descriptors, six QSAR predictive models were developed based on a set of 43 organic compounds, as well as on the total dataset of 54 organic compounds. These six predictive models are suitable for the high-throughput prediction of adsorption energies for organic pollutant onto microplastics in water. The atoms rich in electrons, van der Waals volumes and molecular polarizability exert strong influences on the adsorption of organic pollutants by microplastics in aqueous environments. In addition, modulating the structure of plastics, e.g., by introducing oxygen atoms, can prevent their interactions with organic pollutants, thereby mitigating combined pollution in the environment. This work can give us insights into the adsorption mechanisms on microplastics, which can offer a valuable reference for the design of green plastic alternatives. Notably, the microplastics models in this study was simulated as unaged microplastics with a relatively small size, which may not fully capture the complexity of microplastics in natural environments. As for the aged microplastics in the environment, they are characterized by the presence of different functional groups, such as hydroxyl and carboxyl groups. Therefore, future work should focus on investigating the adsorption on larger microplastic models and those aged variants with specific surface functionalities.

## Figures and Tables

**Table 1:** Aqueous adsorption energies (*E*_ad_) on PE, POM and PVA MPs from DFT computations.

No.	CAS No.	Compound	*E*_ad_ (kcal/mol)		
			PE	POM	PVA
1	100-25-4	1,4-dinitrobenzene	−9.3	−2.76	−6.92
2	100-41-4	ethylbenzene	−9.57	−6.83	−5.29
3	100-47-0	benzonitrile	−7.89	−8.21	−3.47
4	100-51-6	benzyl alcohol	−8.68	−7.99	−9.6
5	103-65-1	n-propylbenzene	−9.53	−7.51	−7.3
6	106-42-3	p-xylene	−8.07	−6.14	−7.21
7	108-39-4	m-cresol	−10.15	−7.18	−6.54
8	108-68-9	3,5-dimethylphenol	−11.22	−7.19	−6.22
9	108-87-2	methylcyclohexane	−8.46	−7.03	−7.1
10	108-88-3	toluene	−9.58	−6.36	−3.24
11	108-95-2	phenol	−8.02	−8.03	−6.48
12	110-82-7	cyclohexane	−7.55	−6.48	−5.89
13	118-79-6	2,4,6-tribromophenol	−54.13	−40.87	−52.16
14	120-12-7	anthracene	−15.35	−7.07	−8.58
15	121-14-2	2,4-dinitrotoluene	−11.55	−3.92	−8.05
16	122-79-2	phenyl acetate	−9.59	−5.95	−5.82
17	123-07-9	4-ethylphenol	−9.49	−9.22	−5.94
18	126-71-6	triisobutyl phosphate	−50.56	−40.86	−47.17
19	129-00-0	pyrene	−16.38	−9.82	−8.83
20	141-82-2	malonic acid	−5.06	−7.79	−12.62
21	207122-16-5	heptabromodiphenyl ether	−59.28	−44.49	−57.25
22	26444-49-5	cresyl diphenyl phosphate	−53.34	−41.23	−47.77
23	3322-93-8	1,2-dibromo-4-(1,2-dibromoethyl)-cyclohexane	−54.22	−42.69	−54.47
24	371-41-5	4-fluorophenol	−7.82	−6.61	−6.81
25	41318-75-6	2,4,4′-tribromodiphenyl ether	−54.36	−38.64	−47.54
26	50-00-0	formaldehyde	−3.65	−3.63	−1.32
27	512-56-1	trimethyl phosphate	−49.29	−38.74	−46.15
28	513-02-0	triisopropyl phosphate	−53.86	−38.07	−48.23
29	528-29-0	1,2-dinitrobenzene	−8.61	−4.92	−3.46
30	585-71-7	(1-bromoethyl)benzene	−50.45	−39.02	−49.56
31	587-03-1	3-methylbenzyl alcohol	−9.54	−7.8	−13.43
32	60-12-8	phenethyl alcohol	−8.87	−9.39	−5.7
33	62-53-3	aniline	−9.41	−9	−6.13
34	64-18-6	formic acid	−2.33	−4.97	−7.11
35	67-66-3	chloroform	−28.06	−23.34	−28.63
36	71-43-2	benzene	−7.68	−4.52	−2.67
37	75-07-0	acetaldehyde	−4.52	−4.52	−0.36
38	78-40-0	triethyl phosphate	−50.09	−40.27	−49.38
39	78-79-5	isoprene	−7.86	−6.24	−4.77
40	791-28-6	triphenyl phosphine oxide	−51.38	−40.22	−49.18
41	813-78-5	dimethyl phosphate	−50.17	−44.24	−52.52
42	85-01-8	phenanthrene	−13.3	−7.38	−8.47
43	86-73-7	fluorene	−13.17	−10.25	−8.32
44	87-82-1	hexabromobenzene	−58.6	−41.97	−55.55
45	90-12-0	1-methylnaphthalene	−12.52	−8.68	−6.24
46	91-20-3	naphthalene	−9.2	−5.16	−6.09
47	923-99-9	tripropyl phosphite	−53.68	−40.51	−48.42
48	92-52-4	biphenyl	−11.84	−7.04	−7.73
49	93-58-3	methyl benzoate	−11.83	−7.16	−5.17
50	93-89-0	ethyl benzoate	−13.14	−7.41	−9.58
51	98-86-2	acetophenone	−11.95	−9.06	−4.1
52	98-95-3	nitrobenzene	−9.41	−2.37	−2.62
53	99-65-0	1,3-dinitrobenzene	−10.82	−4.39	−1.98
54	99-99-0	4-nitrotoluene	−10.29	−2.31	−3.56

**Table 2:** Different predictive models for adsorption onto microplastics in water.

Adsorption Data	Water	Algorithm	*n*_t_	*k*	*R* ^2^	*RMSE*_t_	*Q* ^2^_LOO_	*n*_v_	*R* ^2^_ext_	*Q* ^2^_ext_	*RMSE*_v_	AD	MPs	Ref
log*K*	Seawater	MLR	26	3	0.857	0.880	0.857	11	0.902	0.892	0.752	Y	PE	[27]
log*K*	Freshwater	MLR	17	1	0.896	0.732	0.896	7	0.947	0.910	0.661	Y	PE	[27]
log*K*	Pure water	MLR	47	1	0.811	0.612	0.811	-	-	-	-	Y	PE	[27]
log*K*	Seawater	ANN	26	3	0.988	0.257	-	11	0.988	-	0.236	N	PE	[29]
log*K*	Freshwater	RF	17	1	0.947	0.549	-	7	0.891	-	0.744	N	PE	[29]
log*K*	Pure water	SVM	47	2	0.949	0.356	-	-	0.986	-	0.132	N	PE	[29]
log*K*	Seawater	MLR	26	3	0.907	0.721	0.907	10	0.928	0.923	0.583	Y	PE	[33]
log*K*	Freshwater	MLR	16	2	0.897	0.651	0.897	7	0.934	0.932	0.563	Y	PE	[33]
log*K*	Pure water	MLR	23	2	0.958	0.297	0.958	9	0.994	0.977	0.251	Y	PE	[33]
*E*_ad_	Pure water	MLR	43	4	0.980	2.440	0.980	11	0.920	0.850	7.820	Y	PE	this study
*E*_ad_	Pure water	MLR	54	4	0.960	3.910	0.950	-	-	-	-	Y	PE	this study
*E*_ad_	Pure water	MLR	43	4	0.960	3.110	0.940	11	0.880	0.810	6.590	Y	POM	this study
*E*_ad_	Pure water	MLR	54	4	0.950	3.470	0.930	-	-	-	-	Y	POM	this study
*E*_ad_	Pure water	MLR	43	4	0.960	3.960	0.940	11	0.900	0.850	7.880	Y	PVA	this study
*E*_ad_	Pure water	MLR	54	4	0.940	4.810	0.920	-	-	-	-	Y	PVA	this study

log*K* is the logarithmic value of adsorption equilibrium constant; MLR is multiple linear regression; ANN is artificial neural networks; RF is random forest; SVM is support vector machine; Y/N is yes/no.

## Data Availability

The original contributions presented in this study are included in the article/Supplementary Material. Further inquiries can be directed to the corresponding authors.

## Supplementary Materials

The following supporting information can be downloaded at https://www.mdpi.com/article/10.3390/ma19071403/s1, Table S1: Adsorption equilibrium configuration for the 54 organic compounds on PE, POM and PVA microplastics in aqueous environments; Table S2: Adsorption energies (*E*_ad_) for 15 organic compounds on PE, POM and PVA microplastics from DFT computations under aqueous and gaseous environments; Table S3: *R*^2^ values from 10 random permutations for *E*_ad_ values on PE, POM and PVA MPs; Table S4: Standardized coefficients, *t* and *p* values and variable inflation factor (*VIF*) for the descriptors in the developed QSAR models; Figure S1: Hirshfeld charge analysis for (a) benzene, (b) aniline and (c) nitrobenzene.
