# Supplementary material for: Integrating DFT Computations and QSAR Modeling to Predict Adsorption of Organic Pollutants onto Microplastics in Aqueous Environments
Source: Materials (Basel). 2026 Apr 1;19(7):1403. doi: 10.3390/ma19071403 (PMC13074982; doi:10.3390/ma19071403)
Supplement: Supplementary file 1 [file materials-19-01403-s001.zip › materials-4209017-supplementary.pdf]

Integrating DFT computations and QSAR modelling to predict adsorption of organic pollutants onto microplastics in aqueous environments

Ya Wang,[1*] Chao Li,[2*] Honghong Yi,[1] Xiaolong Tang, [1] Peng Zhao[1]

1. School of Energy and Environmental Engineering, University of Science and Technology Beijing, Beijing 10083, China

2. Engineering Laboratory for Water Pollution Control and Resources Recovery, State Environmental Protection Key Laboratory of Wetland Ecology and Vegetation Restoration, School of Environment, Northeast Normal University, Changchun, 130117, China

Corresponding Authors

* Ya Wang, e-mail: yaanne@126.com (YW)

* Chao Li, e-mail: lic932@nenu.edu.cn (CL)

The supplementary material consists of four tables and one figure. There are 14 pages in total.

Contents

Table S1. Adsorption equilibrium configuration for the 54 organic compounds on PE, POM and PVA microplastics in aqueous environments 4
Table S2. Adsorption energies (E_{ad}) for 15 organic compounds on PE, POM and PVA microplastics from DFT computations under aqueous and gaseous environments 11
Table S3. R^2 values from 10 random permutations for E_{ad} values on PE, POM and PVA MPs 12
Table S4. Standardized coefficients, t, p values and variable inflation factor (VIF) for the descriptors in the developed QSAR models 13
Figure S1. Hirshfeld charge analysis for (a) benzene, (b) aniline and (c) nitrobenzene 14

Table S1. Adsorption equilibrium configuration for the 54 organic compounds on PE, POM and PVA microplastics in aqueous environments

**PE**					
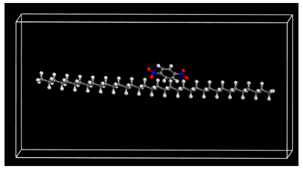<br>*d* = 3.484 Å<br>100-25-4	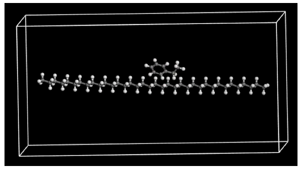<br>*d* = 3.757 Å<br>100-41-4	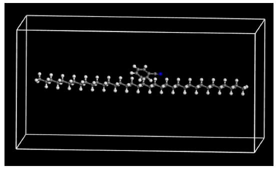<br>*d* = 3.530 Å<br>100-47-0	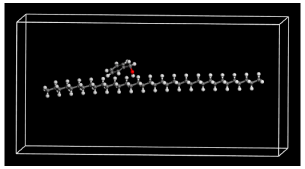<br>*d* = 3.792Å<br>100-51-6	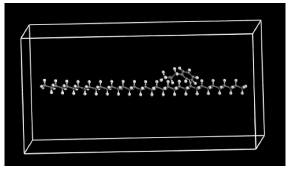<br>*d* = 3.867 Å<br>103-65-1	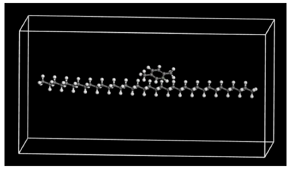<br>*d* = 3.873 Å<br>106-42-3
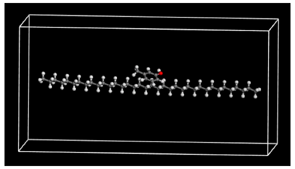<br>*d* = 3.328 Å<br>108-39-4	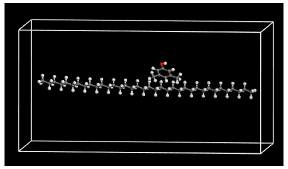<br>*d* = 3.475 Å<br>108-68-9	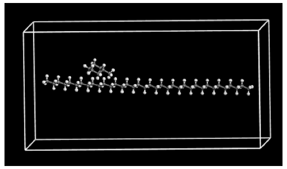<br>*d* = 3.992 Å<br>108-87-2	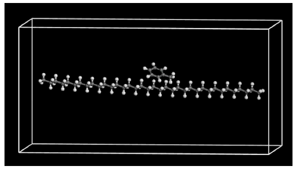<br>*d* = 3.373 Å<br>108-88-3	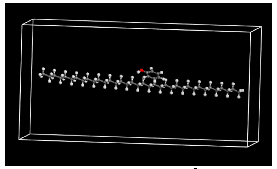<br>*d* = 3.370 Å<br>*108-95-2*	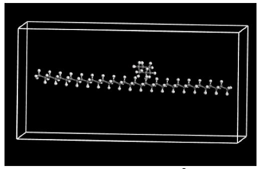<br>*d* = 3.931Å<br>*110-82-7*

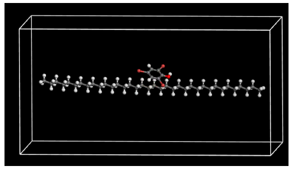<br>d = 3.554 Å<br>118-79-6	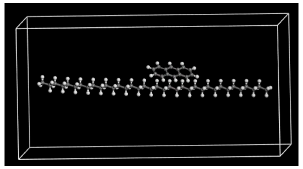<br>d = 3.400 Å<br>120-12-7	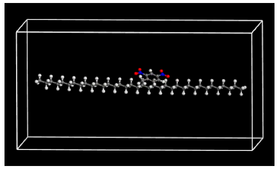<br>d = 3.329Å<br>121-14-2	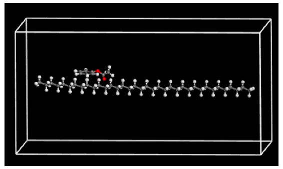<br>d = 3.640 Å<br>122-79-2	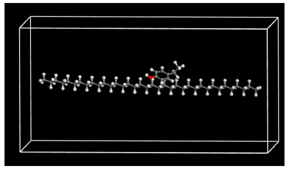<br>d = 3.611 Å<br>123-07-9	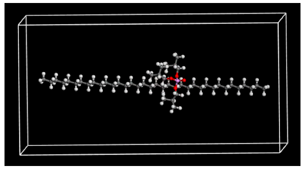<br>d = 3.722 Å<br>126-71-6
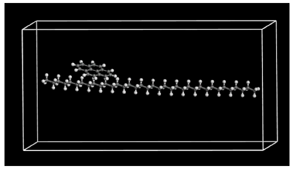<br>d = 3.392 Å<br>129-00-0	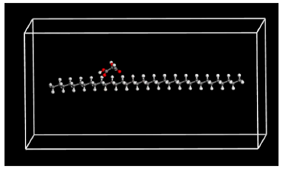<br>d = 3.732 Å<br>141-82-2	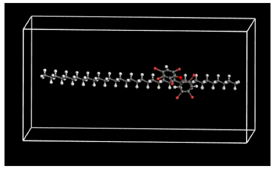<br>d = 3.372 Å<br>207122-16-5	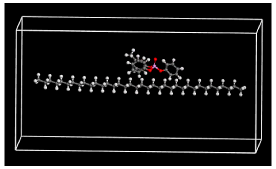<br>d = 4.348 Å<br>26444-49-5	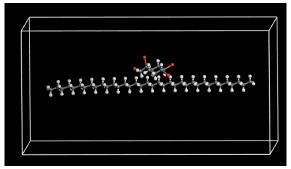<br>d = 4.482 Å<br>*3322-93-8*	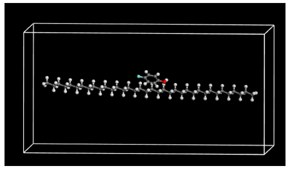<br>d = 3.406 Å<br>*371-41-5*
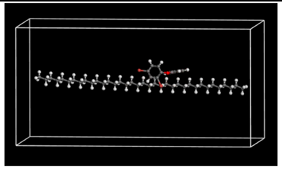<br>d = 3.834 Å<br>41318-75-6	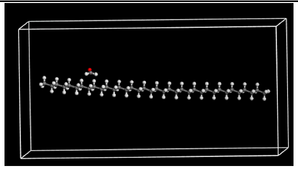<br>d = 3.384 Å<br>50-00-0	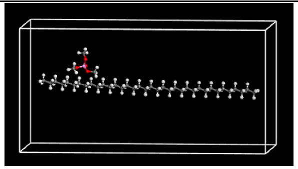<br>d = 3.991 Å<br>512-56-1	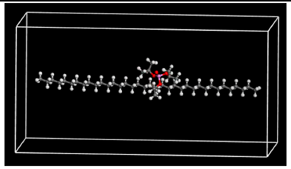<br>d = 3.939 Å<br>513-02-0	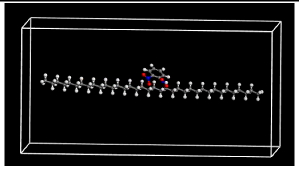<br>d = 3.570 Å<br>*528-29-0*	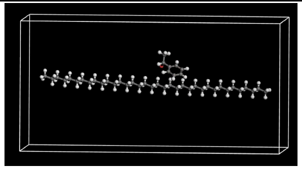<br>d = 3.571 Å<br>*585-71-7*
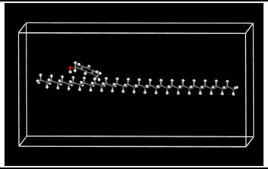	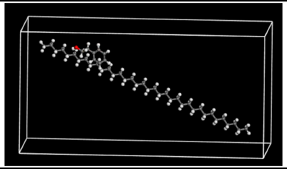	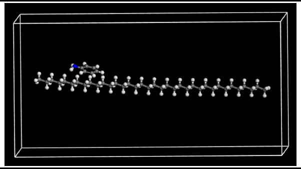	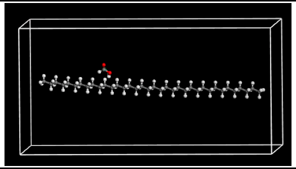	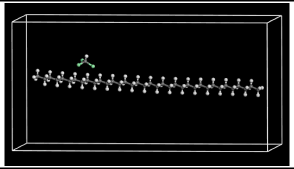	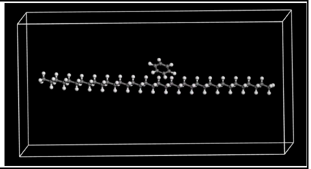

d = 3.719 Å<br>587-03-1	*d* = 3.868 Å<br>60-12-8	*d* = 3.353 Å<br>62-53-3	*d* = 4.044 Å<br>64-18-6	*d* = 4.143 Å<br>*67-66-3*	*d* = 3.376Å<br>*71-43-2*
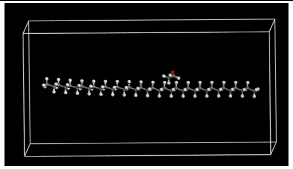<br>*d* = 3.343Å<br>75-07-0	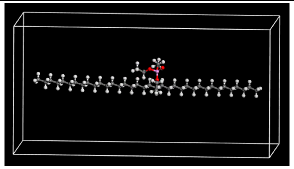<br>*d* = 4.085 Å<br>78-40-0	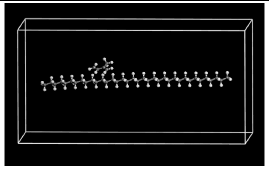<br>*d* = 3.741Å<br>78-79-5	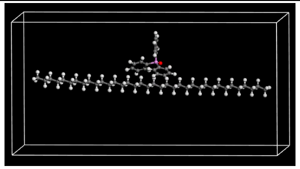<br>*d* = 4.304 Å<br>791-28-6	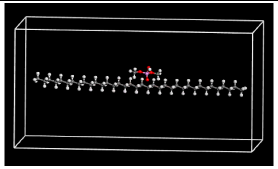<br>*d* = 3.770 Å<br>*813-78-5*	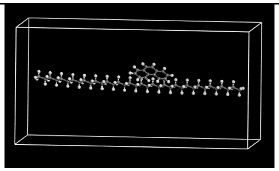<br>*d* = 3.484 Å<br>*85-01-8*
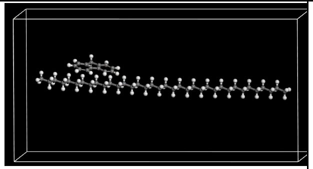<br>*d* = 3.455Å<br>86-73-7	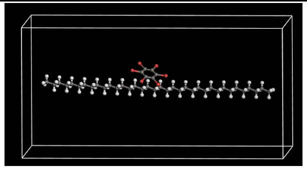<br>*d* = 3.556 Å<br>87-82-1	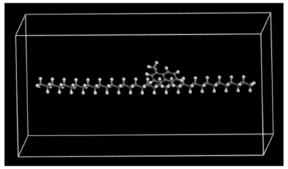<br>*d* = 3.517 Å<br>90-12-0	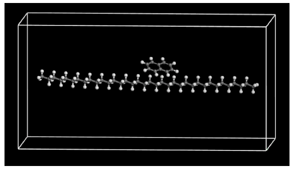<br>*d* = 3.960 Å<br>91-20-3	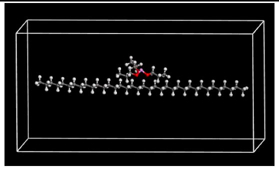<br>*d* = 3.717 Å<br>*923-99-9*	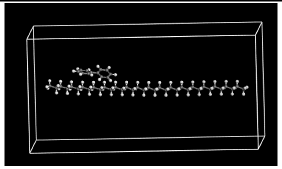<br>*d* = 3.495 Å<br>*92-52-4*
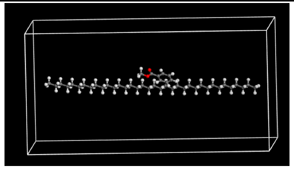<br>*d* = 3.465 Å<br>*93-58-3*	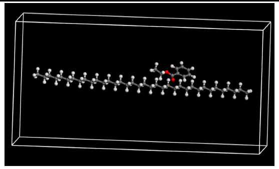<br>*d* = 3.423 Å<br>*93-89-0*	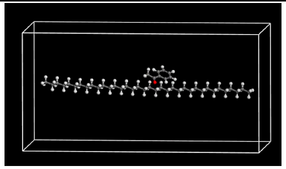<br>*d* = 3.434 Å<br>*98-86-2*	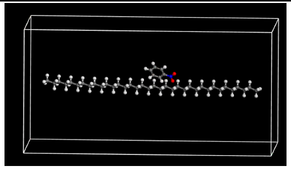<br>*d* = 3.413 Å<br>*98-95-3*	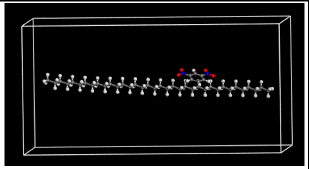<br>*d* = 3.284 Å<br>*99-65-0*	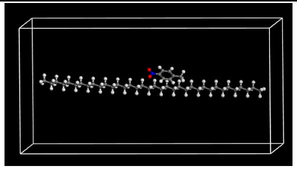<br>*d* = 3.492 Å<br>*99-99-0*
**POM**					
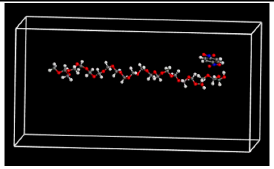<br>*d* = 3.895 Å<br>100-25-4	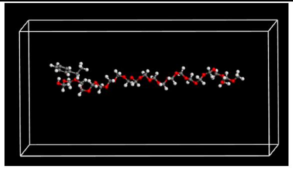<br>*d* = 3.756 Å<br>100-41-4	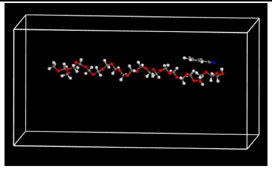<br>*d* = 3.707 Å<br>100-47-0	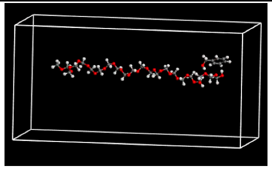<br>*d* = 3.729 Å<br>100-51-6	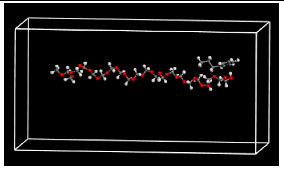<br>*d* = 3.974 Å<br>103-65-1	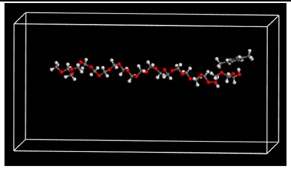<br>*d* =3.788Å<br>106-42-3

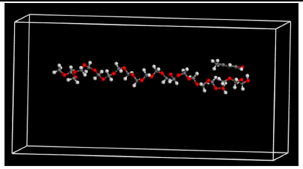<br>*d* = 4.364Å<br>108-39-4	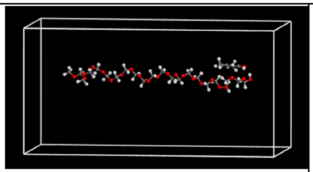<br>*d* = 4.491 Å<br>108-68-9	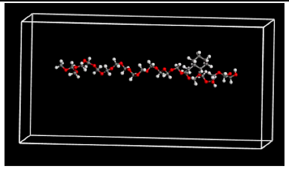<br>*d* = 4.205 Å<br>108-87-2	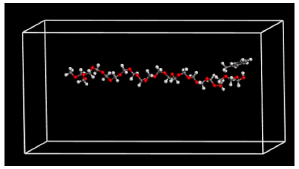<br>*d* = 3.648Å<br>108-88-3	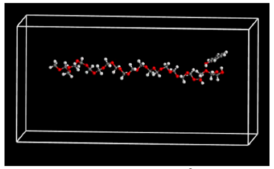<br>*d* = 3.751Å<br>*108-95-2*	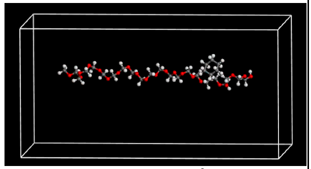<br>*d* = 4.212Å<br>*110-82-7*
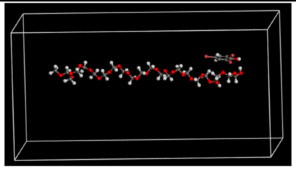<br>*d* = 4.157 Å<br>118-79-6	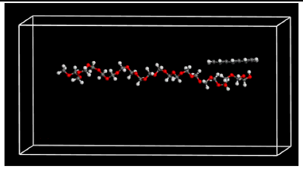<br>*d* = 4.854 Å<br>120-12-7	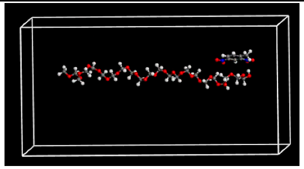<br>*d* = 3.685Å<br>121-14-2	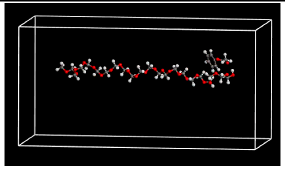<br>*d* = 4.739 Å<br>122-79-2	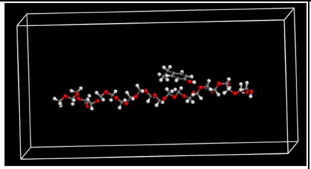<br>*d* = 3.473 Å<br>123-07-9	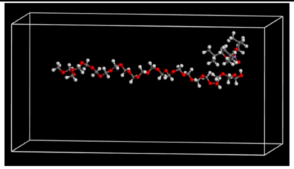<br>*d* = 5.424Å<br>126-71-6
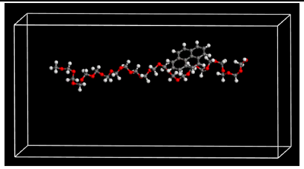<br>*d* = 3.193Å<br>129-00-0	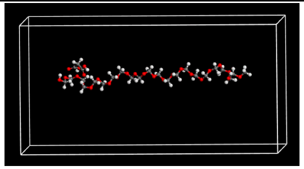<br>*d* = 4.478 Å<br>141-82-2	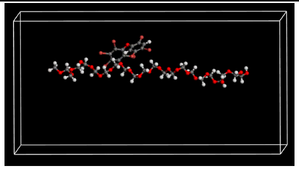<br>*d* = 3.461 Å<br>207122-16-5	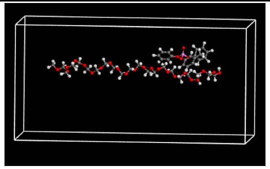<br>*d* = 4.146 Å<br>26444-49-5	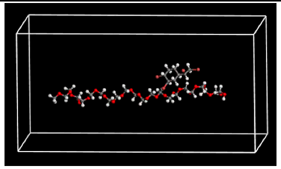<br>*d* =4.608 Å<br>*3322-93-8*	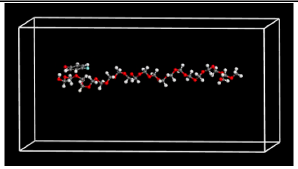<br>*d* = 4.365 Å<br>*371-41-5*
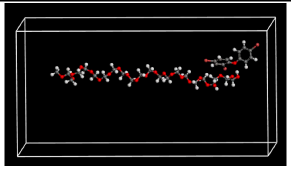<br>*d* = 4.805 Å<br>41318-75-6	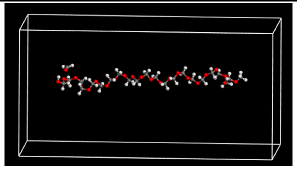<br>*d* = 3.469 Å<br>50-00-0	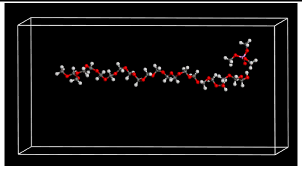<br>*d* = 4.507 Å<br>512-56-1	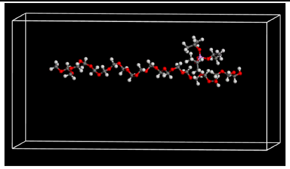<br>*d* = 4.592 Å<br>513-02-0	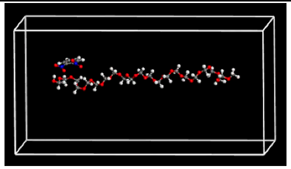<br>*d* = 3.949 Å<br>*528-29-0*	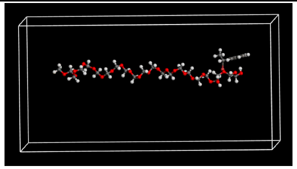<br>*d* = 5.227Å<br>*585-71-7*
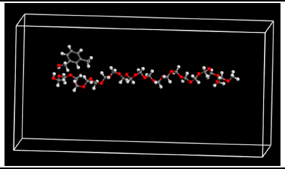	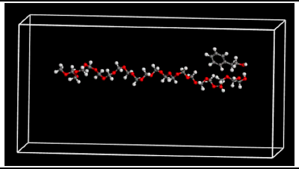	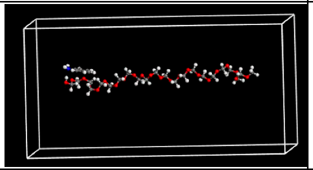	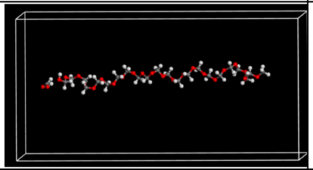	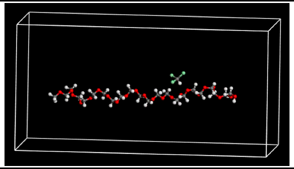	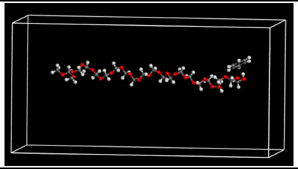

d = 4.271 Å<br>587-03-1	d = 4.874 Å<br>60-12-8	d = 3.922 Å<br>62-53-3	d = 3.617 Å<br>64-18-6	d = 4.152 Å<br>*67-66-3*	d = 3.714 Å<br>*71-43-2*
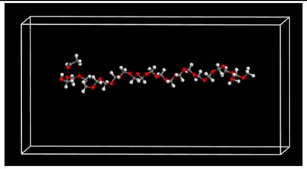<br>d = 3.942 Å<br>75-07-0	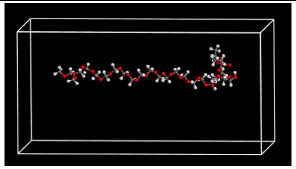<br>d = 4.950 Å<br>78-40-0	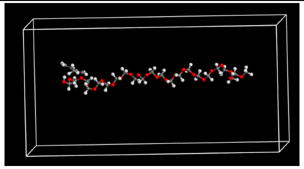<br>d = 4.641 Å<br>78-79-5	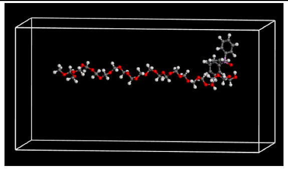<br>d = 5.319 Å<br>791-28-6	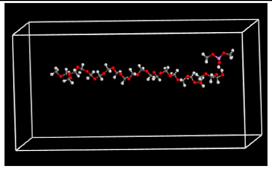<br>d =4.535Å<br>*813-78-5*	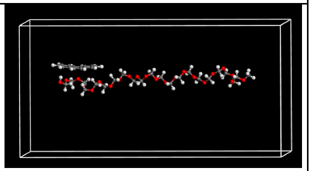<br>d = 4.877 Å<br>*85-01-8*
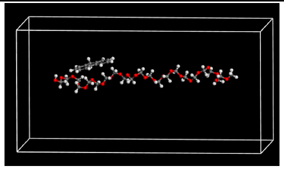<br>d = 3.634 Å<br>86-73-7	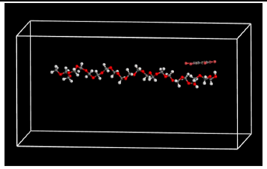<br>d = 3.836 Å<br>87-82-1	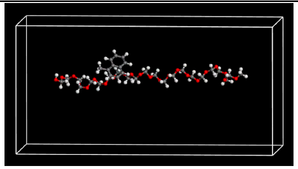<br>d = 3.321 Å<br>90-12-0	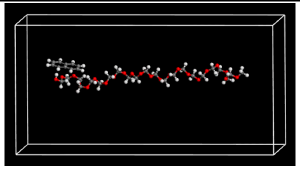<br>d = 4.785 Å<br>91-20-3	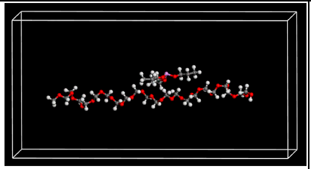<br>d = 3.404 Å<br>*923-99-9*	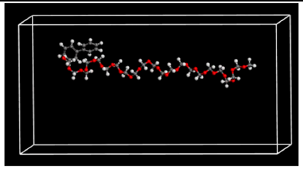<br>d = 4.620 Å<br>*92-52-4*
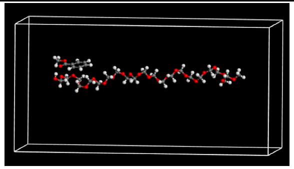<br>d = 4.244 Å<br>*93-58-3*	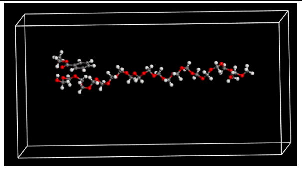<br>d = 4.220 Å<br>*93-89-0*	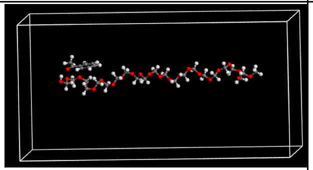<br>d = 4.259 Å<br>*98-86-2*	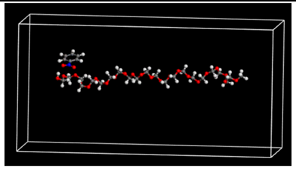<br>d = 3.655 Å<br>*98-95-3*	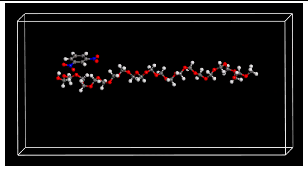<br>d = 3.795 Å<br>*99-65-0*	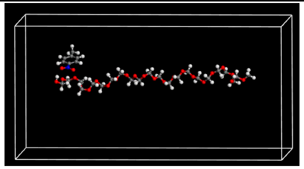<br>d = 3.708 Å<br>*99-99-0*
**PVA**					
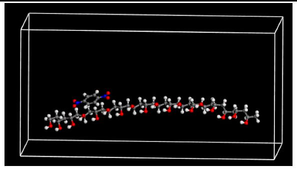<br>d = 3.407 Å<br>100-25-4	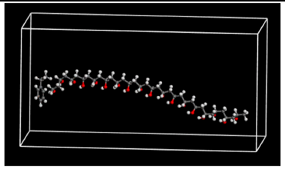<br>d = 3.666 Å<br>100-41-4	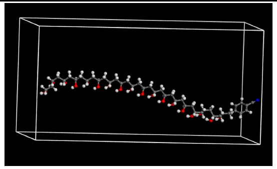<br>d = 5.409 Å<br>100-47-0	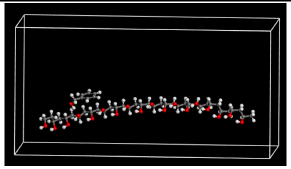<br>d = 3.498 Å<br>100-51-6	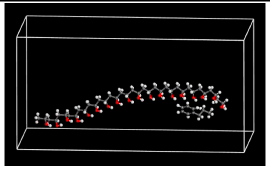<br>d = 4.238 Å<br>103-65-1	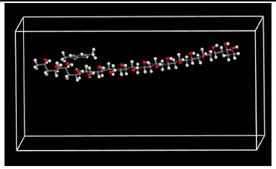<br>d = 3.945 Å<br>106-42-3

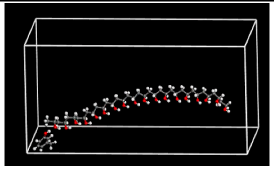<br>*d* = 5.025 Å<br>108-39-4	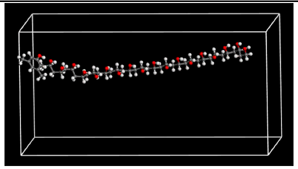<br>*d* = 4.849 Å<br>108-68-9	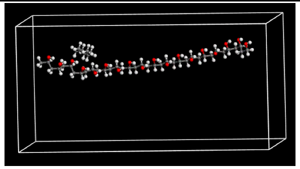<br>*d* = 4.112 Å<br>108-87-2	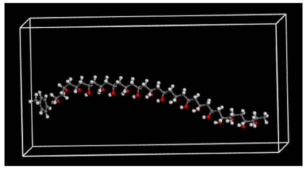<br>*d* = 3.704 Å<br>108-88-3	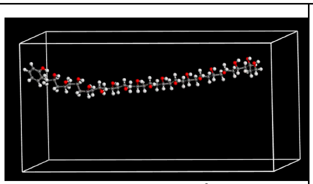<br>*d* = 4.505 Å<br>*108-95-2*	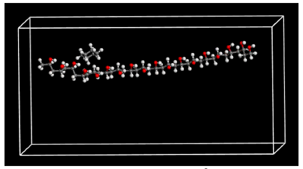<br>*d* = 3.873 Å<br>*110-82-7*
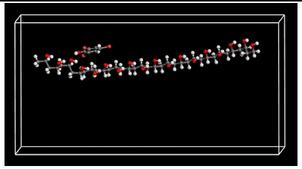<br>*d* = 3.693 Å<br>118-79-6	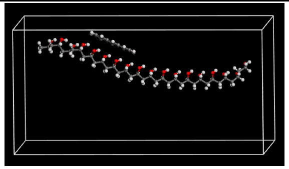<br>*d* = 4.345 Å<br>120-12-7	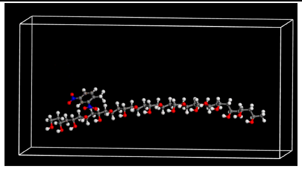<br>*d* = 3.657 Å<br>121-14-2	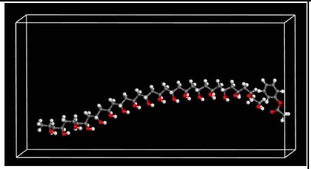<br>*d* = 3.606 Å<br>122-79-2	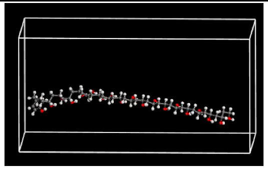<br>*d* = 5.234 Å<br>123-07-9	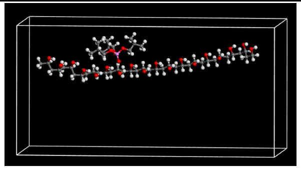<br>*d* = 4.582 Å<br>126-71-6
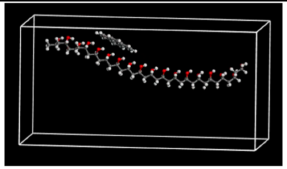<br>*d* = 4.298 Å<br>129-00-0	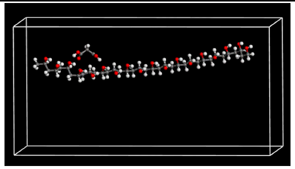<br>*d* = 3.534 Å<br>141-82-2	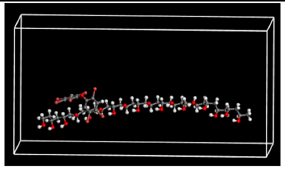<br>*d* = 3.936 Å<br>207122-16-5	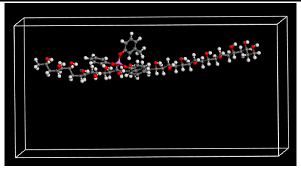<br>*d* = 5.155 Å<br>26444-49-5	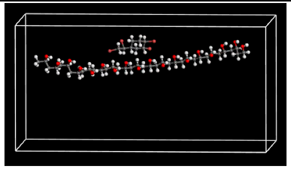<br>*d* = 4.475 Å<br>*3322-93-8*	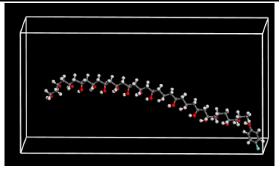<br>*d* = 4.889 Å<br>*371-41-5*
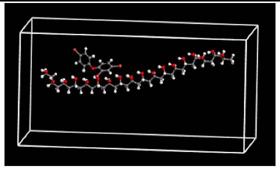<br>*d* = 4.911 Å<br>41318-75-6	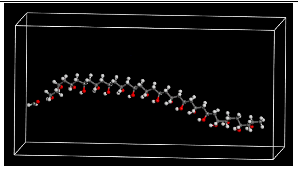<br>*d* = 4.474 Å<br>50-00-0	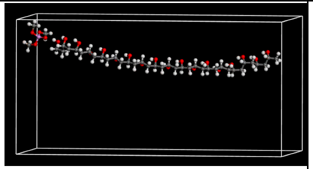<br>*d* = 5.035 Å<br>512-56-1	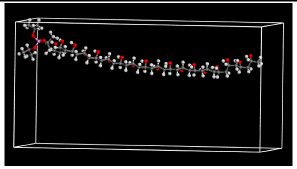<br>*d* = 5.014 Å<br>513-02-0	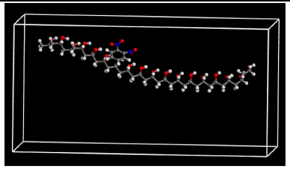<br>*d* = 4.279 Å<br>*528-29-0*	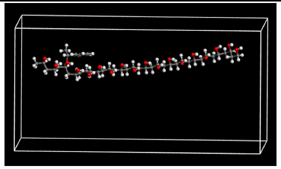<br>*d* = 3.805 Å<br>*585-71-7*
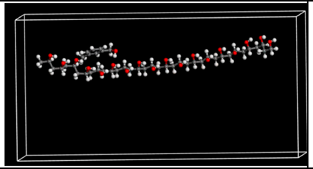	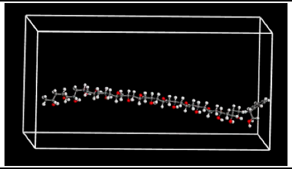	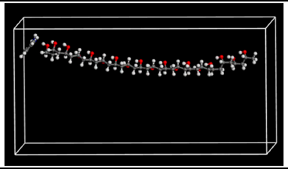	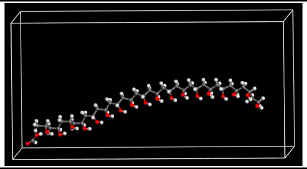	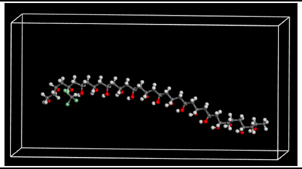	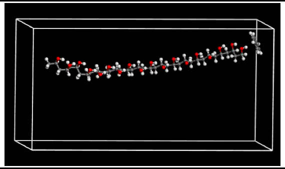

d = 3.488 Å<br>587-03-1	*d* = 5.210 Å<br>60-12-8	*d* = 3.626 Å<br>62-53-3	*d* = 3.672 Å<br>64-18-6	*d* = 4.003 Å<br>*67-66-3*	*d* = 3.876 Å<br>*71-43-2*
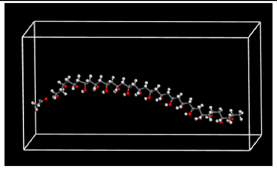<br>*d* = 4.394 Å<br>75-07-0	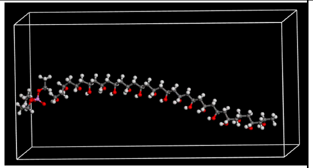<br>*d* = 4.647 Å<br>78-40-0	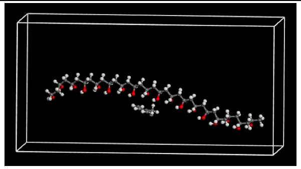<br>*d* = 4.093 Å<br>78-79-5	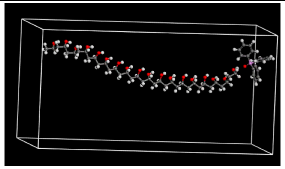<br>*d* = 4.663 Å<br>791-28-6	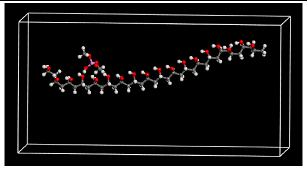<br>*d* = 4.103 Å<br>*813-78-5*	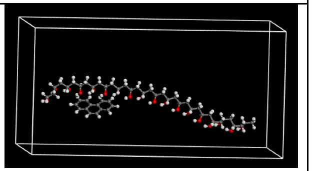<br>*d* = 4.334 Å<br>*85-01-8*
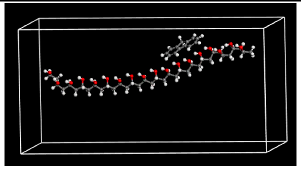<br>*d* = 4.318 Å<br>86-73-7	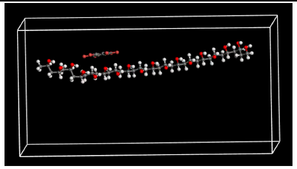<br>*d* = 3.988 Å<br>87-82-1	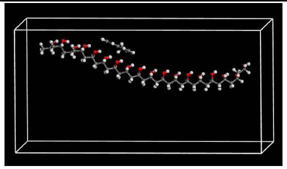<br>*d* = 4.477 Å<br>90-12-0	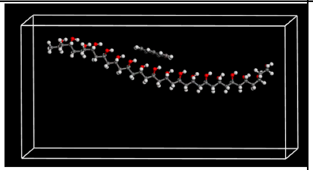<br>*d* = 4.416 Å<br>91-20-3	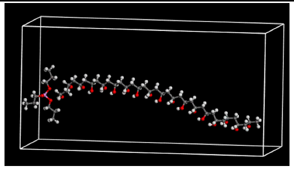<br>*d* = 3.706 Å<br>*923-99-9*	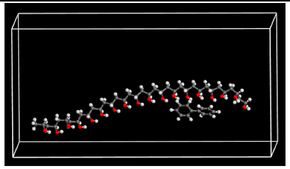<br>*d* = 4.330 Å<br>*92-52-4*
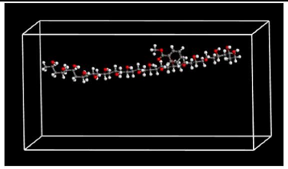<br>*d* = 4.169 Å<br>*93-58-3*	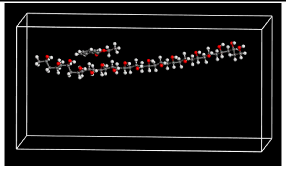<br>*d* = 3.709 Å<br>*93-89-0*	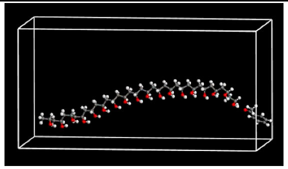<br>*d* = 5.493 Å<br>*98-86-2*	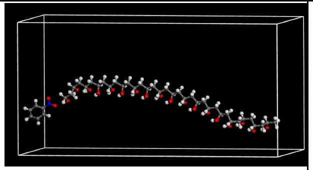<br>*d* = 5.423 Å<br>*98-95-3*	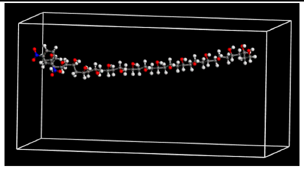<br>*d* = 3.892 Å<br>*99-65-0*	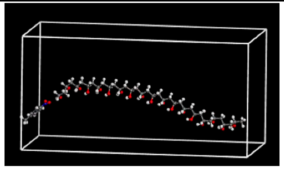<br>*d* = 5.499 Å<br>*99-99-0*

d represents the distance between the center of molecule and the nearest carbon atom in PE, POM or PVA microplastics.

Table S2. Adsorption energies (E_{ad}) for 15 organic compounds on PE, POM and PVA microplastics from DFT computations under aqueous and gaseous environments

No.	Compound	E_{ad} (aqueous phase) (kcal/mol)			E_{ad} (gaseous phase) (kcal/mol)		
		PE	POM	PVA	PE	POM	PVA
1	formaldehyde	–3.65	–3.63	–1.32	–3.12	–9.51	–7.22
2	acetaldehyde	–4.52	–4.52	–0.36	–3.68	–10.51	–7.24
3	formic acid	–2.33	–4.97	–7.11	–5.91	–9.66	–15.89
4	malonic acid	–5.06	–7.79	–12.62	–6.96	–16.42	–23.65
5	cyclohexane	–7.55	–6.48	–5.89	–6.37	–8.18	–8.52
6	methylcyclohexane	–8.46	–7.03	–7.1	–7.17	–8.68	–9.89
7	benzene	–7.68	–4.52	–2.67	–7.36	–8.54	–7.44
8	toluene	–9.58	–6.36	–3.24	–9.36	–10.69	–8.12
9	aniline	–9.41	–9	–6.13	–10.06	–13.80	–11.65
10	phenol	–8.02	–8.03	–6.48	–8.67	–15.62	–12.83
11	nitrobenzene	–9.41	–2.37	–2.62	–8.86	–8.20	–10.41
12	benzonitrile	–7.89	–8.21	–3.47	–7.65	–13.24	–11.90
13	benzyl alcohol	–8.68	–7.99	–9.6	–9.79	–14.81	–16.11
14	ethylbenzene	–9.57	–6.83	–5.29	–9.14	–11.15	–10.24
15	n-propylbenzene	–9.53	–7.51	–7.3	–9.04	–11.67	–11.15

Table S3. R^2 values from 10 random permutations for E_{ad} values on PE, POM and PVA MPs

No.	$R^2(n = 43)$			$R^2(n = 54)$		
	PE	POM	PVA	PE	POM	PVA
1	0.25	0.12	0.07	0.02	0.12	0.08
2	0.04	0.12	0.13	0.00	0.14	0.03
3	0.08	0.13	0.09	0.20	0.11	0.04
4	0.19	0.03	0.03	0.08	0.11	0.02
5	0.12	0.10	0.10	0.09	0.17	0.09
6	0.05	0.04	0.06	0.12	0.09	0.02
7	0.10	0.14	0.10	0.09	0.06	0.08
8	0.06	0.11	0.07	0.05	0.02	0.05
9	0.11	0.12	0.00	0.09	0.07	0.03
10	0.22	0.06	0.05	0.18	0.03	0.07
Mean	0.12	0.10	0.07	0.09	0.09	0.05

Table S4. Standardized coefficients, *t*, *p* values and variable inflation factor (*VIF*) for the descriptors in the developed QSAR models

QSAR models with 43 organic compounds												
	PE				POM				PVA			
Descriptors	Standardized coefficients	*t**	*p**	*VIF*	Standardized coefficients	*t**	*p**	*VIF*	Standardized coefficients	*t**	*p**	*VIF*
ATSC1m	–0.420	–8.315	< 0.001	5.916	–0.552	–6.764	< 0.001	5.916	–0.506	–6.269	< 0.001	5.916
AATSC0v	–0.091	–3.938	< 0.001	1.238	–0.118	–3.153	< 0.01	1.238	–0.066	–1.795	< 0.1	1.238
MATS1m	–0.173	–3.520	< 0.01	5.567	–0.037	–0.469	< 1	5.567	–0.047	–0.594	< 1	5.567
BCUTw-1h	–0.693	–30.119	< 0.001	1.224	–0.661	–17.810	< 0.001	1.224	–0.706	–19.221	< 0.001	1.224
QSAR models with 54 organic compounds												
	PE				POM				PVA			
Descriptors	Standardized coefficients	*t**	*p**	*VIF*	Standardized coefficients	*t**	*p**	*VIF*	Standardized coefficients	*t**	*p**	*VIF*
AATS1m	–0.405	–13.207	< 0.001	1.122	–0.386	–11.130	< 0.001	1.122	–0.427	–11.488	< 0.001	1.122
AATS7p	0.261	5.375	< 0.001	2.796	0.288	5.263	< 0.001	2.796	0.257	4.369	< 0.001	2.796
ATSC0p	–0.903	–17.260	< 0.001	3.255	–0.899	–15.206	< 0.001	3.255	–0.857	–13.524	< 0.001	3.255
AATSC1p	0.122	3.573	< 0.01	1.380	0.173	4.482	< 0.001	1.380	0.154	3.731	< 0.001	1.380

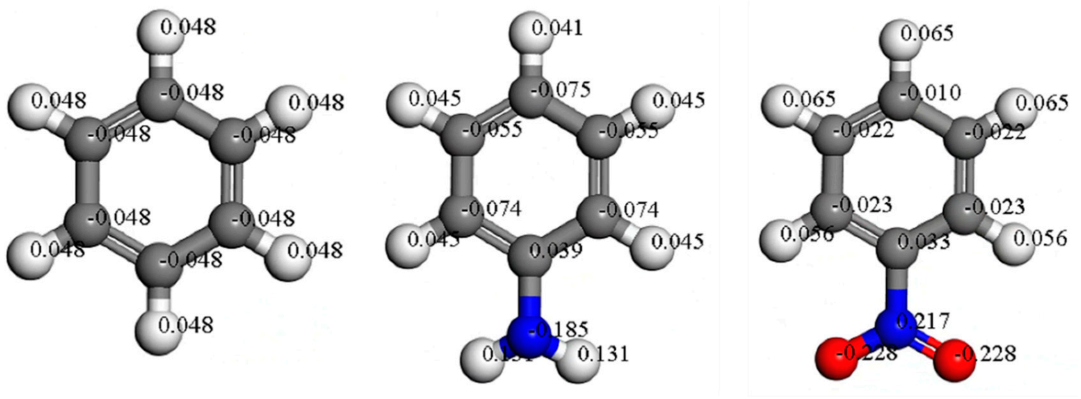

(a) benzene (b) aniline (c) nitrobenzene

Figure S1. Hirshfeld charge analysis for (a) benzene, (b) aniline and (c) nitrobenzene
